# Association of Cerebrovascular and Alzheimer Disease Biomarkers With Cholinergic White Matter Degeneration in Cognitively Unimpaired Individuals

**DOI:** 10.1212/WNL.0000000000200930

**Published:** 2022-10-11

**Authors:** Nira Cedres, Daniel Ferreira, Milan Nemy, Alejandra Machado, Joana B. Pereira, Sara Shams, Lars-Olof Wahlund, Anna Zettergren, Olga Stepankova, Lenka Vyslouzilova, Maria Eriksdotter, Stefan Teipel, Michel J. Grothe, Kaj Blennow, Henrik Zetterberg, Michael Schöll, Silke Kern, Ingmar Skoog, Eric Westman

**Affiliations:** From the Division of Clinical Geriatrics (N.C., D.F., A.M., J.B.P., S.S., L.-O.W., M.E., E.W.), Centre for Alzheimer Research, Department of Neurobiology, Care Sciences, and Society, and the Division of Insurance Medicine (A.M.), Department of Clinical Neuroscience, Karolinska Institutet, Stockholm; Department of Psychology (N.C.), Sensory Cognitive Interaction Laboratory (SCI-lab), Stockholm University, Sweden; Department of Radiology (D.F.), Mayo Clinic, Rochester, MN; Department of Cybernetics (M.N.), Faculty of Electrical Engineering and Czech Institute of Informatics (M.N., O.S., L.V.), Robotics, and Cybernetics, Czech Technical University, Prague, Czech Republic; Clinical Memory Research Unit (J.B.P.), Department of Clinical Sciences, Lund University, Malmö; Department of Psychiatry Cognition and Old Age Psychiatry, (A.Z., S.K., I.S.), Clinical Neurochemistry Laboratory (K.B., H.Z.), and Department of Clinical Physiology (M.S.), Sahlgrenska University Hospital, Gothenburg; Neuropsychiatric Epidemiology Unit (A.Z., K.B., H.Z., S.K., I.S.), Department of Psychiatry and Neurochemistry, Institute of Neuroscience and Physiology, the Sahlgrenska Academy, Centre for Ageing and Health (AGECAP) at the University of Gothenburg; Theme Inflammation and Aging (M.E.), Karolinska University Hospital, Huddinge, Sweden; Clinical Dementia Research Section (S.T.), Department of Psychosomatic Medicine, University Medicine Rostock; German Center for Neurodegenerative Diseases (DZNE) (S.T., M.J.G.), Rostock, Germany; Unidad de Trastornos del Movimiento (M.J.G.), Servicio de Neurología y Neurofisiología Clínica, Instituto de Biomedicina de Sevilla (IBiS), Hospital Universitario Virgen del Rocío/CSIC/Universidad de Sevilla, Spain; Department of Neurodegenerative Disease (H.Z.), UCL Institute of Neurology, London; Dementia Research Institute at UCL (H.Z.), London, UK; Hong Kong Center for Neurodegenerative Diseases (H.Z.), China; Wallenberg Centre for Molecular and Translational Medicine (M.S.) and Department of Psychiatry and Neurochemistry (M.S.), Institute of Physiology and Neuroscience, University of Gothenburg, Sweden; Dementia Research Centre (M.S.), Institute of Neurology, University College London; and Department of Neuroimaging (E.W.), Centre for Neuroimaging Sciences, Institute of Psychiatry, Psychology and Neuroscience, King's College London, UK.

## Abstract

**Background and Objectives:**

Several pathologic processes might contribute to the degeneration of the cholinergic system in aging. We aimed to determine the contribution of amyloid, tau, and cerebrovascular biomarkers toward the degeneration of cholinergic white matter (WM) projections in cognitively unimpaired individuals.

**Methods:**

The contribution of amyloid and tau pathology was assessed through CSF levels of the Aβ_42/40_ ratio and phosphorylated tau (p-tau). CSF Aβ_38_ levels were also measured. Cerebrovascular pathology was assessed using automatic segmentations of WM lesions (WMLs) on MRI. Cholinergic WM projections (i.e., cingulum and external capsule pathways) were modeled using tractography based on diffusion tensor imaging data. Sex and APOE ε4 carriership were also included in the analysis as variables of interest.

**Results:**

We included 203 cognitively unimpaired individuals from the H70 Gothenburg Birth Cohort Studies (all individuals aged 70 years, 51% female). WM lesion burden was the most important contributor to the degeneration of both cholinergic pathways (increase in mean square error [IncMSE] = 98.8% in the external capsule pathway and IncMSE = 93.3% in the cingulum pathway). Levels of Aβ_38_ and p-tau also contributed to cholinergic WM degeneration, especially in the external capsule pathway (IncMSE = 28.4% and IncMSE = 23.4%, respectively). The Aβ_42/40_ ratio did not contribute notably to the models (IncMSE<3.0%). APOE ε4 carriers showed poorer integrity in the cingulum pathway (IncMSE = 21.33%). Women showed poorer integrity of the external capsule pathway (IncMSE = 21.55%), which was independent of amyloid status as reflected by the nonsignificant differences in integrity when comparing amyloid-positive vs amyloid-negative women participants (T_201_ = −1.55; *p* = 0.123).

**Discussion:**

In cognitively unimpaired older individuals, WMLs play a central role in the degeneration of cholinergic pathways. Our findings highlight the importance of WM lesion burden in the elderly population, which should be considered in the development of prevention programs for neurodegeneration and cognitive impairment.

The cholinergic neurons located in the nucleus basalis of Meynert (NBM) provide the major cholinergic input to the cerebral cortex and are essential to cognitive functioning.^1^ Postmortem studies have traced 2 principal cholinergic projection pathways from the NBM to the neocortex: the medial and the lateral pathways.^1^ The medial pathway advances through the white matter (WM) axons of the rectus gyrus, bends at the rostrum of the corpus callosum, and enters the cingulum bundle, projecting to the paraolfactory, cingulate, and retrosplenial cortices. The lateral pathway advances both through the claustrum and the extreme capsule (i.e., perisylvian division), projecting to the frontoparietal operculum, insula, and superior temporal gyrus, and through the external capsule and uncinate fasciculus (i.e., capsular division), projecting to the remaining parts of the frontal, parietal, and temporal neocortex. Recent diffusion tensor imaging (DTI)-based tractography studies have examined these pathways,^2–5^ providing the opportunity to study the integrity of the cholinergic system and its potential association with cognitive performance and pathophysiologic processes in vivo.

The strategic location of the NBM and its connective circuitry to the cortex results in increased vulnerability to brain pathology. For example, cholinergic neurons are affected in early stages of Alzheimer disease (AD)-related tauopathy due to their proximity to heavily affected basotemporal regions, which likely also alters their connective circuitry to the cortex.^1^ Furthermore, other age-related pathologies can also affect the integrity of the cholinergic system. WM lesions (WMLs), which are thought to be a marker of cerebrovascular disease, are commonly found on MRI in the elderly.^6^ A recent study showed that WMLs are associated with worse integrity of the cholinergic projections in cognitively unimpaired older individuals,^4^ and cholinergic projections influenced cognitive performance.^4^ Of interest, despite the association of WMLs with the integrity of the cholinergic projection system, neither WML burden itself nor NBM volume contributed to cognitive performance.^4^ These findings raised the question of whether other age-associated pathologies apart from WMLs might be affecting the integrity of the cholinergic projections in cognitively unimpaired individuals.

In this study, we investigated the contribution of amyloid and tau pathology in combination with cerebrovascular disease toward the degeneration of cholinergic WM projections in cognitively unimpaired individuals. It is important to address these research questions to assess whether and how other pathologies apart from cerebrovascular disease may affect the integrity of cholinergic projections in cognitively unimpaired individuals.

## Methods

### Participants

The study sample belongs to the Gothenburg H70 Birth Cohort Studies.^7^ Every 70-year-old listed in the Swedish Population Registry as a resident in Gothenburg (Sweden) was invited to a comprehensive examination on aging and age-related factors.^7^ A total of 1,203 individuals born in 1944 (response rate 72.2%; mean age 70.5 years) agreed to participate, of whom 430 consented to a lumbar puncture (response rate 35.8%). Lumbar puncture was considered as contraindicated in participants under anticoagulant therapy, immune-modulated therapy, and cancer therapy. After excluding participants not suitable for a lumbar puncture, the CSF extraction was conducted in 322 (26.8%) individuals. Every participant was also invited to take part in a brain MRI examination, of which 792 individuals (response rate 65.8%) underwent MRI conducted at Aleris in Gothenburg. The MRI examination was conducted within 3 months from the initial study visit. The lumbar puncture was conducted within 2 months from the MRI examination. The general examinations and other procedures have previously been described in detail.^7^ General cognitive status was measured using the Mini-Mental State Examination (MMSE) and the Clinical Dementia Rating (CDR) scale. For the current study, inclusion criteria were (1) a CDR score of 0; (2) MMSE >24; (3) availability of CSF biomarkers; and (4) availability of MRI data, yielding a final sample of 203 individuals (51% female).

### MRI Data Acquisition, Image Processing, and Assessment of WMLs

MRI data were acquired in a 3.0 T Philips Achieva system (Philips Medical Systems), using a 3D T1-weighted turbo field echo sequence (repetition time [RT] = 7.2 ms, echo time [TE] = 3.2 ms, flip angle = 9°, matrix size = 250 × 250 mm, field of view = 256 × 256, and slice thickness = 1.0 mm); a 3D Fluid-attenuated inversion recovery (FLAIR) sequence (RT = 48,000 ms, TE = 280 ms, TI = 1,650 ms, flip angle = 90°, number of slices = 140, matrix size = 250 × 237 mm, and slice thickness = 2.0 mm); a susceptibility-weighted imaging (SWI) sequence (RT = 14.59–17.60 ms, TE = 20.59–24.99 ms, flip angle = 10°, matrix size = 229 × 222 mm, and slice thickness = 1.0 mm); and a DTI sequence encoded with 1 b-value shell: 800 ks/mm^2^, along with 32 directions and 1 b = 0 image (RT = 7,340 ms, TE = 83 ms, flip angle = 90°, matrix size = 112 × 112 mm, field of view = 224 × 224, and slice thickness = 3.0 mm).^7^

WMLs were measured as WM hypointensities and WM hyperintensities in T1-weighted and FLAIR sequences, respectively. WML and total intracranial volume (TIV) were automatically segmented using FreeSurfer 6.0.0. FreeSurfer detects hypointense WM signal abnormalities and automatically labels WML volumes for each participant using a probabilistic procedure.^8^ Hyperintense WMLs were automatically segmented using the open source segmentation toolbox LST 2.0.15.^9^ It has previously been shown that hypointense and hyperintense WMLs are strongly correlated.^6^ Previous findings revealed that hypointense WMLs might represent necrotic damage closer to accumulated cerebrovascular pathology,^10^ whereas hyperintense WMLs might also represent acute damage including peri-inflammatory processes.^11^ Due to the aim of the current study, we focused on hypointense WMLs, but all the analyses were replicated using hyperintense WMLs and are reported in eFigure 1, links.lww.com/WNL/C220. MRI data management and processing was performed using theHiveDB^12^ database system. WML volumes in milliliters (mL) were adjusted by TIV to account for variability in head size.^13^

Previously established ROI masks for the cholinergic WM pathways (i.e., cingulum and external capsule pathways) were used.^4^ Briefly, the masks were created using probabilistic diffusion-based fiber tracking of the NBM WM projections. These ROI masks of the cholinergic WM pathways were transferred from MNI standard space to each individual DTI image (b0) in native space using the nonlinear SyN registration algorithm^14^ from advanced normalization tools.^15^ Native space mean diffusivity (MD) maps were calculated for each subject using the FMRIB Diffusion Toolbox from FSL.^16^ Microstructural properties of each participant's cholinergic WM tracts were then calculated by averaging the MD values within the back-transformed ROI masks in native space. The MD index was preferred over the fractional anisotropy (FA) index because MD is more robust in the influence of crossing fibers.^17^

### Complementary MRI Markers of Cerebrovascular Disease and Vascular Risk Factors

In addition to the automated measure of WMLs,^18^ we assessed cerebral microbleeds, lacunes, and superficial siderosis for completeness of information. The presence/absence of cerebral microbleeds was visually assessed on SWI, lacunes (3–15 mm) were assessed on FLAIR images, and superficial siderosis was assessed on SWI. All visual assessments were performed by an experience neuroradiologist blinded to clinical data,^19^ according to the Standards for Reporting Vascular Changes on Neuroimaging and standard scales and standardized scales.^20^ We also recorded and described the frequency of vascular risk factors, including hypertension, diabetes, smoking, and ischemia as assessed through a semistructured interview and clinical examination by research nurses or medical doctors.^7^

### CSF Sampling and Biomarker Analysis

Lumbar puncture for CSF sampling and determination of *APOE* e4 carriership were conducted following standard procedures.^7^ CSF biomarker levels were determined by a commercially available assay.^7^ CSF tau phosphorylated at threonine 181 (p-tau) was determined by immunoassay ELISA (INNOTEST PHOSPHO_TAU [181P]). The Aβ_42/40_ ratio and CSF Aβ_38_ were determined by the V-PLEX Aβ Peptide Panel 1 (6E10) Kit (Meso Scale Discovery, Rockville, MD). We used p-tau to assess tau neurofibrillary tangle pathology. The CSF Aβ_42/40_ ratio was used as a marker of amyloidosis.^21^ For descriptive purposes, each individual was classified as positive (+; i.e., abnormal) or negative (−; i.e., normal) according to CSF biomarkers for Aβ (CSF Aβ_42_) and p-tau (CSF p-tau) following cohort-specific cutoff values: ≤530 pg/mL for Aβ_42_ and p-tau >80.^22^ Aβ_38_, a shorter isoform of Aβ that can also be found in the CSF, is still poorly understood. A previous study suggested that Aβ_38_ could be a marker of AD.^23^ Another study reported a predominant localization of Aβ_38_ within the vascular vessels in patients with AD.^24^ In addition, there is also evidence showing the presence of Aβ_38_ in other non-AD dementias^25–27^ and patients with chronic neuroinflammation.^23^ These diverse findings reflect the view that the role of Aβ_38_ still needs to be elucidated. Hence, we included CSF Aβ_38_ in this study to determine its association with AD biomarkers and cerebrovascular disease in the general population.

### Statistical Analysis

Statistical analyses were conducted using R statistical software.^28^ A *p* value <0.05 (2 tailed) was deemed significant in all the analyses.

We used random forest (RF) regression models to assess the differential contributions of the different pathology-specific biomarkers toward the integrity of NBM projections. Two separate RF regression models, treated as the outcome variables, were fitted for the prediction of MD in the cingulum and the external capsule pathway, respectively. MD values were multiplied by a constant (c = 10,000) to facilitate the visualization of the data. WML, CSF Aβ_42/40_ ratio, Aβ_38_, and p-tau were included as predictors in all RF models, along with sex (i.e., male/female) and *APOE* status (i.e., at least 1 ε4 allele to be treated as carrier, otherwise noncarrier). RF is a machine learning method that estimates multiple decision trees via bootstrap aggregation (bagging). Each tree predicts a classification independently and votes for the corresponding class. The majority of the votes decide the overall prediction.^29,30^ A conditional importance score is computed for each tree in RF analysis. This is performed by measuring the change in the prediction error when the values of a certain variable are permuted within a grid defined by the included covariates. Then, this conditional score is averaged across the entire ensemble. These conditional importance scores are designed to reduce the undesirable effects of collinearity among predictor variables. The final importance of each predictor denotes its contribution to the model. Importance values below or equal to zero denote no contribution. A conditional regression tree is produced as a graphical representation of the model. The RF comprised 5,000 conditional inference trees. R2 was computed to assess the quality of the RF models. Although aging is associated with WM neurodegeneration and greater WML volumes,^4,31^ age was not included as a covariate in the models because it was controlled from the design (i.e., all participants were aged 70 years). For completeness of information, we also report Pearson correlation coefficients among the predictor variables included in the RF models and independent sample *t* tests for categorical variables that resulted important in the RF analysis. The randomForest^32^ and party packages^33^ were used for these analyses.

### Standard Protocol Approvals, Registrations, and Patient Consents

The H70 study was approved by the Regional Ethical Review Board in Gothenburg (Approval Numbers: 869-13, T076-14, T166-14, 976-13, 127-14, T936-15, 006-14, T703-14, 006-14, T201-17, T915-14, 959-15, and T139-15) and by the Radiation Protection Committee (Approval Number: 13-64) in concordance with the 1964 Helsinki Declaration and its later amendment.

### Data Availability

The authors state that anonymized data on which the article is based will be shared by request from any qualified investigator.

## Results

Demographic, clinical data, vascular risk factors, and MRI markers of cerebrovascular disease are shown in Table 1. In our sample of 203 cognitively unimpaired individuals (all aged 70 years, 51% female), 2% had an AD biomarker profile (i.e., A+ T+), 43% had abnormal CSF levels of β-amyloid only (i.e., A+ T−), and 4.4% had abnormal CSF levels of p-tau only (i.e., A− T+). Results are shown for hypointense WML volume from T1-weighted 3D images. Virtually, the same results were obtained when including hyperintense WMLs instead of hypointense WMLs in the models (eFigure 1, links.lww.com/WNL/C220).

**Table 1:**
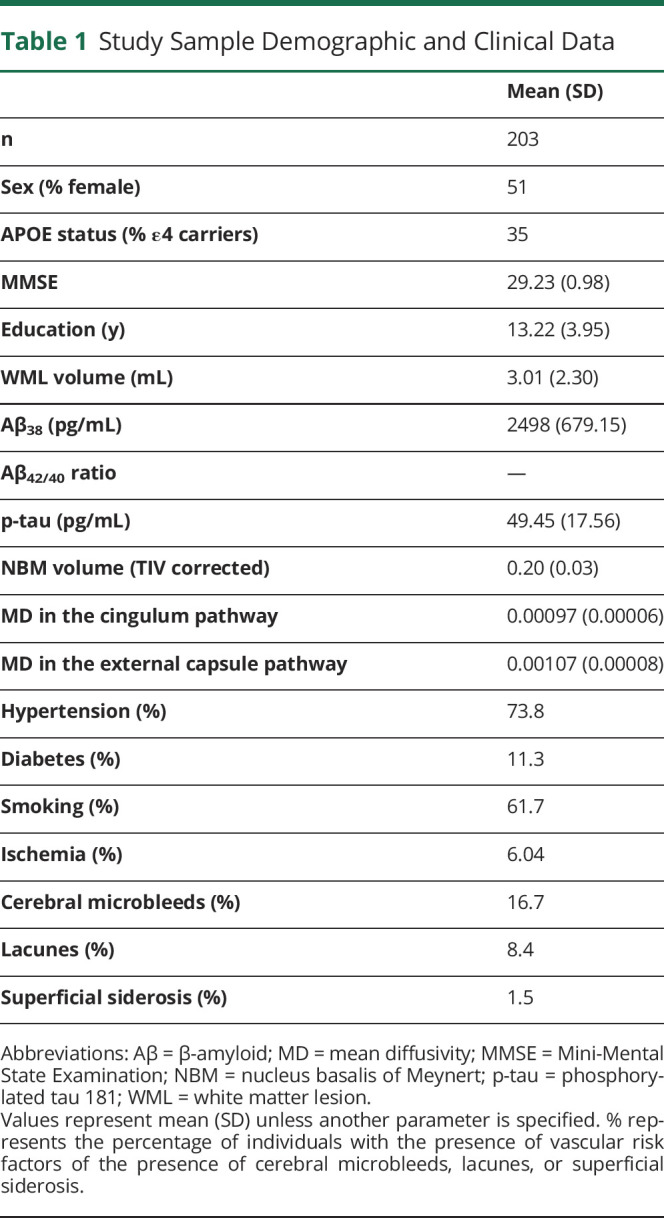
Study Sample Demographic and Clinical Data

**Figure 1:**
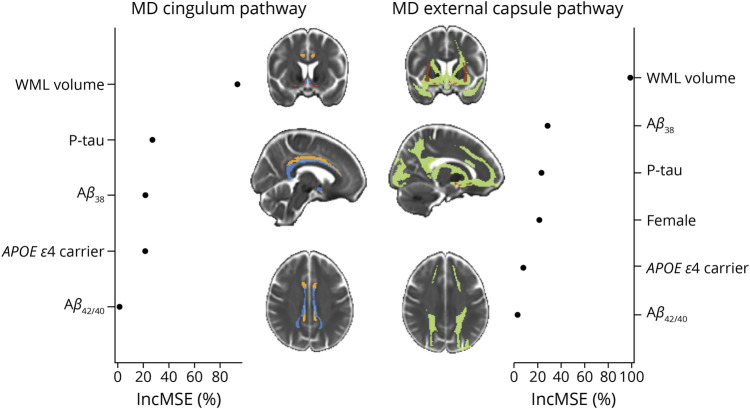
Contribution of Amyloid, Tau, and Cerebrovascular Biomarkers Toward the Integrity of Cholinergic WM Pathways The plot represents the percentage of increase in the prediction error (%IncMSE) when removing each variable from the random forest model. NBM ROI is represented in red. The cingulum pathway is represented in blue (orange for the cingulum mask). The external capsule pathway is represented in green (brown for the external capsule mask). Aβ_38_ = amyloid β_38_; Aβ_42/40_ = amyloid β_42/40_ ratio; P-tau = phosphorylated tau; WML volume = white matter lesions based on hypointense signal abnormalities of T1-weighted 3D images.

**Figure 2:**
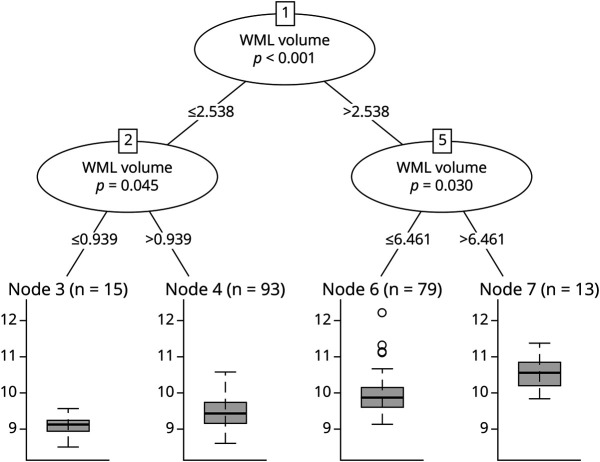
Random Forest Regression Tree for the MD in the Cingulum Pathway The figure represents the recursive partitioning for the MD index in the cingulum pathway and the contribution of WML volume to the portioning. Corresponding *p* values are given for each inner node. Boxplot for MD distribution is shown for each final group. MD = mean diffusivity; WML volume = white matter lesions based on hypointense signal abnormalities of T1-weighted 3D images.

The RF models showed that WML volume was the most important predictor for the average MD of the cingulum pathway (Figure 1). P-tau, Aβ_38_, and *APOE* ε4 carriership were also important predictors in the model. The Aβ_42/40_ ratio received a low importance score. Sex did not contribute to the MD in the cingulum pathway. The RF tree revealed that WML volume was the best predictor splitting individuals according to their MD in the cingulum pathway. Four groups were distinguished (Figure 2). P-tau, Aβ_38_, Aβ_42/40_ ratio, sex, and *APOE* ε4 carriership did not separate any of the groups based on their association with MD in the cingulum pathway.

**Figure 3:**
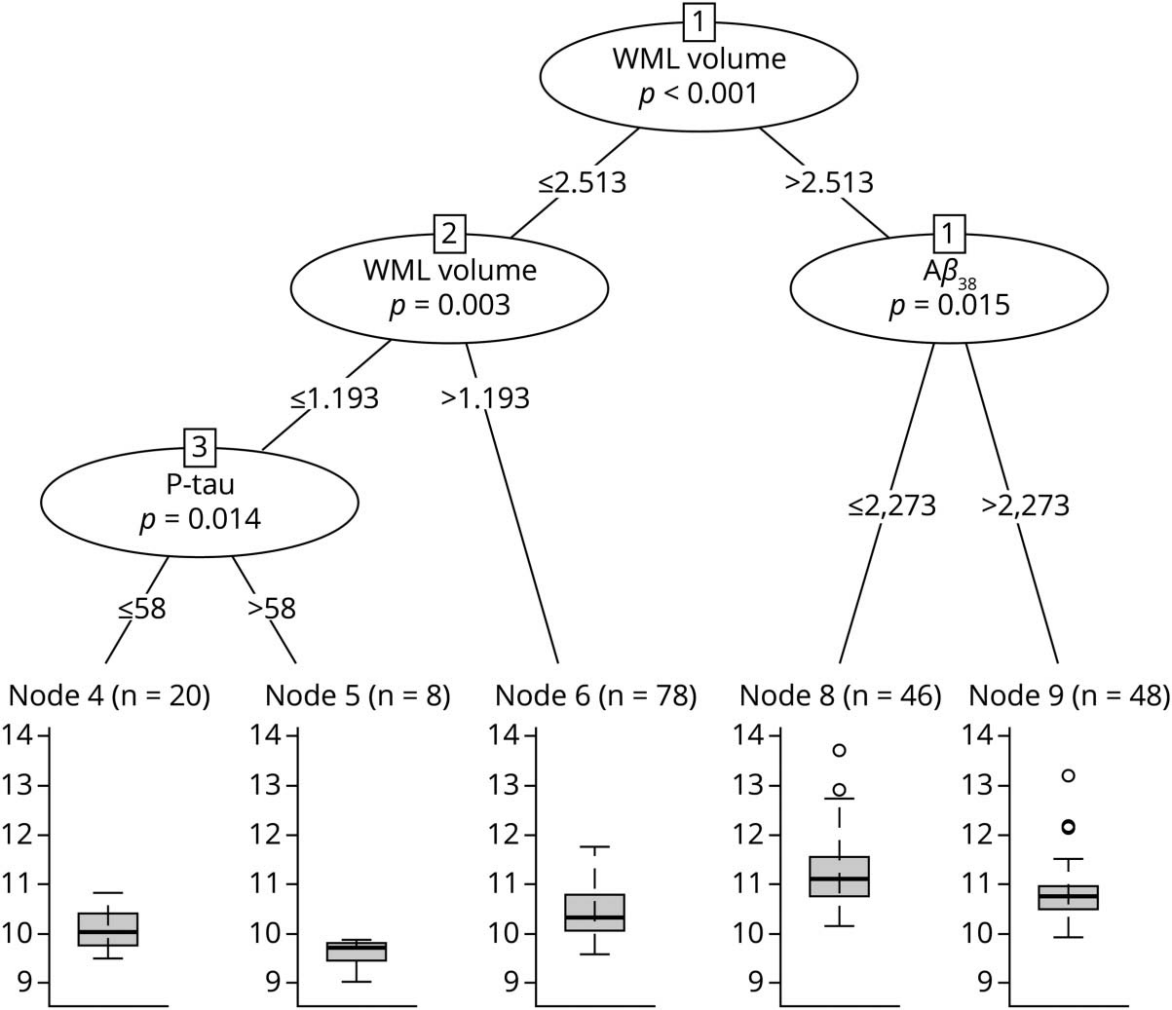
Random Forest Regression Tree for the MD in the External Capsule Pathway The figure represents the recursive partitioning for the MD index in the external capsule pathway and the contribution of WML volume, p-tau, and Aβ_38_ to the portioning. Corresponding *p* values are given for each inner node. Boxplot for MD distribution is shown for each final group. Aβ_38_ = amyloid β_38_; MD = mean diffusivity; P-tau = phosphorylated tau; WML volume = white matter lesions based on hypointense signal abnormalities of T1-weighted 3D images.

Regarding the prediction of the MD in the external capsule pathway, WML volume was again the most important predictor (Figure 1). Aβ_38_, p-tau, and sex were also important in the model. Women showed poorer integrity in the external capsule pathway. This finding was independent of amyloid status, as reflected by the nonsignificant differences in integrity when comparing amyloid-positive vs amyloid-negative women participants (T_201_ = −1.55; *p* = 0.123). *APOE* ε4 carriership received a low importance score, and the Aβ_42/40_ ratio did not contribute to the MD in the external capsule pathway. The RF tree revealed that WML volume, Aβ_38_, and p-tau were important predictors to split individuals according to their MD in the external capsule. Five groups were distinguished at the end of the tree (Figure 3).

**Figure 4:**
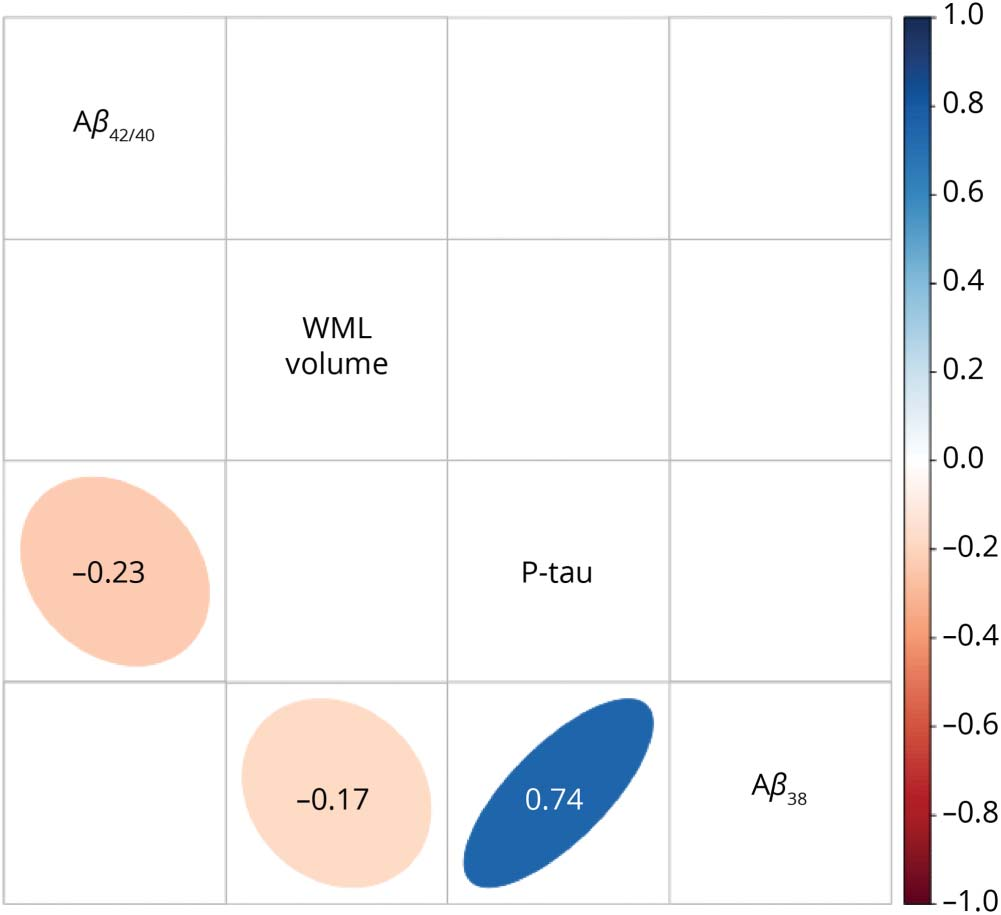
Correlation Matrix for the Predictors Included in the Random Forest Models Background of significant correlations (*p* < 0.05) was colored according to the value of the correlation coefficient and shaped accordingly to the association distribution, otherwise left empty.

Figure 4 shows the correlation matrix for all pairs of continuous predictors in the RF models. Greater WML volumes were associated with lower Aβ_38_ levels. Higher p-tau levels were associated with lower Aβ_42/40_ ratio and higher Aβ_38_ levels.

## Discussion

In our study, we investigated the contribution of cerebrovascular disease compared with amyloid pathology and tau pathology toward the degeneration of cholinergic WM pathways in cognitively unimpaired individuals. We demonstrated the role of WML burden as a central contributor to the degeneration of the cholinergic projections.

The NBM is well known for its key role in cognitive functioning and its deterioration is linked to cognitive impairment in AD.^1^ It is important to determine the pathologic processes contributing toward degeneration of the cholinergic system as it has previously been demonstrated to be associated with cognitive impairment in advanced aging.^4^ In this sample of cognitively unimpaired aged individuals, we demonstrated that WMLs were the most important contributor toward the degeneration of the studied cholinergic pathways, followed by CSF Aβ_38_ and p-tau levels. Conversely, the Aβ_42/40_ ratio did not show a substantial contribution.

The integrity of the cholinergic system is crucial for proper cognitive functioning.^1^ The cholinergic hypothesis of cognitive aging postulates that age-related memory decline and other cognitive problems may arise due to declining cholinergic activity.^34,35^ In a previous study, we demonstrated that the WM integrity of cholinergic projections was closely associated with attention and memory performance in an independent aging cohort of cognitively unimpaired individuals.^4^ The influence of WML burden on cortical disconnection of the cholinergic system might be associated with subclinical cognitive impairments in the elderly. Longitudinal studies have shown that a high WML burden increases the risk of future cognitive impairment.^36^ Future studies should determine the disruptive role of WMLs in the association between cholinergic projections and cognitive performance in normal aging and the continuum of AD.

Although WML burden was the most important predictor in our RF models, we found that Aβ_38_ also contributed to the integrity of the cholinergic system. In contrast, the Aβ_42/40_ ratio was not an important predictor of neurodegeneration of cholinergic WM projections. The role of CSF Aβ_38_ and its association with neurodegeneration is still under debate.^37,38^ CSF Aβ_38_ levels are lower in frontotemporal dementia^25^ and dementia with Lewy bodies^26,27^ than in patients with AD. Furthermore, Aβ_38_ has previously been linked to increased counts of lacunes and cerebral microbleeds, 2 markers of cerebrovascular disease.^39^ Deposits of Aβ_38_ in vascular vessels have also been found in postmortem AD studies.^24^ Therefore, several studies suggest a potential association of Aβ_38_ with cerebrovascular pathology. In line with this, we showed that lower CSF Aβ_38_ levels were associated with a higher WML burden. In our study, both WML burden and CSF Aβ_38_ were the most important predictors of WM neurodegeneration of the cholinergic system compared with AD biomarkers (CSF Aβ_42/40_ and p-tau). These findings suggest an association between Aβ_38_ and cerebrovascular disease in normal aging and their predilection for the cholinergic WM. Recent reports have demonstrated that higher levels of Aβ38 in the CSF may have a protective effect against future cognitive decline and AD dementia in individuals with a positive AD biomarker profile at baseline.^40^ In support of this, decreased CSF Aβ_38_ levels have previously been linked to reduced cingulate and insula cortex volumes in our cohort.^37^ The cingulate cortex receives important cholinergic input from the medial cholinergic pathway and the insula from the lateral cholinergic pathway.^1^ These areas are well known for their role in emotion regulation, behavior, and executive functioning.^41^ Future studies should test whether Aβ_38_, neurodegeneration of the cholinergic system and reduced cingulate and insula gray matter volumes are associated with subclinical changes in emotion regulation and executive functioning in the elderly.

The cholinergic circuitry is highly vulnerable to brain pathology. In our study, we found pathway-dependent associations of WML, Aβ_38_, and tau (p-tau) pathologic markers with cholinergic WM projections. Our results show that individuals with decreased Aβ_38_ and high WML burden had the poorest integrity of the external capsule pathway. Of interest, women also showed poorer integrity in the external capsule pathway, independently of amyloid status. In contrast, WML burden was the only predictor of the integrity in the cingulum pathway. These pathway-dependent findings point to a greater vulnerability of the cingulum pathway to vascular pathology, in comparison to amyloid/tau pathologies. Regionally, the cingulum pathway is located in periventricular regions, where the presence of WMLs increases with aging.^42^ Periventricular WMLs have previously been associated with lower cortical cholinergic activity in normal aging.^43^ Conversely, the external capsule pathway might be more vulnerable to cerebrovascular disease and pathologies associated with Aβ_38_.

Regarding tau pathology, our results showed a negative association between p-tau and degeneration of cholinergic WM projections (i.e., a poorer integrity of WM projections was associated with lower levels of CSF p-tau). This counterintuitive finding might be the result of a selection bias in our sample. All our participants were cognitively unimpaired 70-year-olds, and only 6.4% had abnormal CSF p-tau levels. It is important to take into consideration that the combination of abnormal levels of p-tau with other brain pathologies such as WMLs will most probably result in cognitive impairment, and therefore, those individuals may have been excluded from our study. Whether increased CSF p-tau levels are associated with degeneration of cholinergic WM projections needs to be further tested in more diverse populations of older individuals, including patients with cognitive impairment.

The data provided by this study describe the contribution of the CSF Aβ_42/40_ ratio, Aβ_38_, and p-tau levels in combination with WML burden toward the degeneration of the cholinergic system in cognitively unimpaired elderly from a population-based cohort.^7^ However, all individuals included were aged 70 years; therefore, results can only be partially generalized to other age groups. A limitation of the current study, intrinsic to the tractography approach used to generate the cholinergic WM projection masks, is the existence of transverse crossing WM fibers that can lead to distorted information about the WM integrity. We aimed to partly overcome this limitation by using the MD index instead of FA because MD is less affected by crossing fibers.^17^ The associations between amyloid/tau biomarkers and WMLs might lead to collinearity problems. Using RF regression with conditional inference trees, we were able to handle multicollinearity to some degree. Alternative information about the spatial location of WMLs and cholinergic functional activity profiles based on fMRI could complement the findings of our current study.^2^ We demonstrated an association between Aβ38 and the degeneration of the cholinergic system. Nevertheless, the literature about the role of Aβ38 in neurodegenerative processes is still limited, and further research is needed. There is currently a discussion ongoing as to whether the validated biomarker cutoffs for dementia diagnosis are clinically relevant for preclinical stages of the disease.^44^ Subthreshold pathology in individuals exhibiting normal biomarker profiles might already be affecting the brain integrity leading to WM degeneration. Thus, in our study, we used continuous values as the input for the analysis. The integrity of the cholinergic projections across abnormal amyloid/tau profiles in clinical stages of AD needs to be further elucidated. Finally, a previous study demonstrated that WMLs can also be related to AD pathology.^45^ However, in our study, WMLs were not associated with CSF levels of Aβ_42/40_ and p-tau, which suggests that our WML measure likely does not reflect AD pathology.^20^

This study highlights the importance of cerebrovascular pathology relative to amyloid and tau pathology in their contribution to cholinergic neurodegeneration in cognitively unimpaired individuals. WMLs within cholinergic pathways correlate with cognitive impairment^46^ and executive dysfunction^47^ in patients with dementia. Given the central role of the cholinergic system in cognition, our study suggests that management of cholinergic WMLs and vascular risk factors should be considered in the development of prevention programs for neurodegeneration and cognitive impairment. As these data are replicated in independent cohorts, it may help in clinical considerations with regard to cerebrovascular and AD biomarkers, cholinergic dysfunction, and cognitive impairment. This knowledge could eventually support therapeutic decisions in the context of acetylcholinesterase inhibitors.

## Glossary

AD: Alzheimer disease
CDR: Clinical Dementia Rating
DTI: diffusion tensor imaging
FA: fractional anisotropy
FLAIR: Fluid-attenuated inversion recovery
MD: mean diffusivity
MMSE: Mini-Mental State Examination
NBM: nucleus basalis of Meynert
RF: random forest
RT: repetition time
SWI: susceptibility-weighted imaging
TE: echo time
TIV: total intracranial volume
WM: white matter
WML: WM lesion
